# Real-Time Processing of Two-Photon Calcium Imaging Data Including Lateral Motion Artifact Correction

**DOI:** 10.3389/fninf.2018.00098

**Published:** 2018-12-18

**Authors:** Akinori Mitani, Takaki Komiyama

**Affiliations:** Neurobiology Section, Center for Neural Circuits and Behavior and Department of Neurosciences, University of California, San Diego, La Jolla, CA, United States

**Keywords:** two-photon calcium imaging, real-time image processing, image registration, closed-loop experiments, motion artifacts

## Abstract

Two-photon calcium imaging has been extensively used to record neural activity in the brain. It has been long used solely with *post-hoc* analysis, but the recent efforts began to include closed-loop experiments. Closed-loop experiments pose new challenges because they require fast, real-time image processing without iterative parameter tuning. When imaging awake animals, one of the crucial steps of *post hoc* image analysis is correction of lateral motion artifacts. In most of the closed-loop experiments, this step has not been implemented and ignored due to technical difficulties. We recently reported the first experiments with real-time processing of calcium imaging that included lateral motion correction. Here, we report the details of the implementation of fast motion correction and present performance analysis across several algorithms with different parameters. Additionally, we introduce a novel method to estimate baseline calcium signal using kernel density estimate, which reduces the number of parameters to be tuned. Combined, we propose a novel software pipeline of real-time image processing suited for closed-loop experiments. The pipeline is also useful for rapid *post hoc* image processing.

## Introduction

Two-photon calcium imaging has been widely used to image the activity of neurons in awake behaving animals. Neurons are loaded with a calcium-sensitive dye or, more commonly, made to express a genetically encoded calcium indicator, such that their fluorescence signal reflects spiking activity of the neurons. However, neural activity is not the sole cause of fluorescence signal change. The recorded movie often entails some artifacts, such as motion artifacts caused by body movements of the awake behaving animals and baseline changes often observed during continuous imaging. To better infer neural activity from the fluorescence signal, it is crucial to correct these artifacts. Of motion artifacts, lateral motion can be computationally corrected. As such, lateral motion correction has been a crucial step in processing calcium images from awake behaving animals. After motion artifact is removed, regions of interest (ROIs) are defined, and average fluorescence intensity of the pixels in each ROI is calculated. Ratio of calcium fluorescence transient to estimated baseline is calculated to infer the spiking activity of the cell. This process of taking the ratio can also make the inference less sensitive to gradual shift of ROIs if not corrected by lateral motion correction. In real-time closed loop experiments, all the image processing steps must be performed on each frame with minimal delay, and parameters cannot be tuned iteratively by assessing the outcome. If parameters depend on imaging conditions, it must be tuned and set at the beginning of the experiment, and failure of selecting the right parameters would result in invalidating the experiment. Therefore, a fast, integrated pipeline to remove those artifacts with a small number of parameters is a prerequisite for real-time closed loop experiments.

TurboReg (Thévenaz et al., 1998) has been widely used to correct lateral motion artifacts. It utilizes a pyramid approach, and it first constructs an image pyramid of series of downscaled images. The transformation at the final resolution is obtained by optimization using a transformation estimated with a downscaled image as an initial value, and this step is repeated recursively several times. A caveat of image downscaling is that when an image is downscaled too much, the process can remove fine spatial features important for motion correction.

Two-photon microscopes generally use the excitation light to scan across the sample, and movement during scanning can not only shift but also distort the image, because each pixel or line is scanned at a different time. Several methods based on hidden Markov model (Dombeck et al., 2007) and optical flow (Greenberg and Kerr, 2009) have been reported to correct distortion caused in applications with low scanning rate.

TurboReg can correct motion artifacts with transformation up to four landmarks, but it could be beneficial to align more than four landmarks when imaging a larger field of view. Recently, NoRMCorre (Pnevmatikakis and Giovannucci, 2017) has been reported to estimate non-rigid transformation for such application. This method splits imaging field into overlapping patches, estimates translation of each patch, and upsamples the dislocation to obtain translation at each pixel. This method requires each patch to contain enough spatial signal to enable frame-by-frame alignment, and may not be applicable when labeling is sparse or weak. If some patches do not contain enough spatial features, the alignment of the patch may be unstable and affects the registration of nearby pixels. Scanbox, a Matlab-based imaging software, includes an automatic stabilization feature by aligning multiple manually selected subregions in real time (Ringach^1^), but the implementation detail and performance analysis have not been published.

These previous motion correction methods are generally so slow that it can be an analytical bottleneck. Thus, efforts have been made to improve the speed of motion correction. For instance, moco is a fast motion correction algorithm based on discrete Fourier transform and cache-aware upsampling, achieving faster motion correction than TurboReg (Dubbs et al., 2016). Similar to TurboReg, moco minimizes L2 distance between the template image and the corrected image normalized by the area of the overlap from all possible pixel-by-pixel shifts. It was written in Java as an ImageJ plugin and reported to have close to real-time performance in *post hoc* analysis. While moco can only estimate translation of images, non-rigid transformation may not be necessary with high scanning rate that has become more common with resonant scanners, because it can make within-frame distortion negligible. In fact, translation-based motion correction algorithms have been widely used in *post hoc* analysis, although it may be problematic with higher zoom that can be affected by small within-frame displacements. In this study, we focused on rigid translation correction.

Discrete Fourier transform-based registration corrects motion artifact up to pixel-by-pixel accuracy. When each ROI only contains a small number of pixels, subpixel registration can potentially improve accuracy of estimating the calcium signal. Registering upsampled image is an approach to achieve subpixel accuracy, but it increases computation for registration. An efficient method has been introduced to only calculate upsampled correlation coefficients around the optimal pixel-by-pixel shift (Guizar-Sicairos et al., 2008). This can be done without fully calculating the inverse discrete Fourier transform of the upsampled images, thus reducing the memory requirement and computation time. However, it has been reported that the overall registration accuracy was lower compared to moco or TurboReg when applied to images from two-photon calcium imaging (Dubbs et al., 2016).

Algorithms discussed above are intensity-based registration algorithms. Alternatively, feature-based registration can be used to correct motion artifact (Aghayee et al., 2017). This can be beneficial when the features are easily recognizable in each frame, but it may fail when signal to noise ratio is low. At the time of the research, the implementation was not available^2^ (empty repository, accessed on 7/8/2018) and in this study we focused on intensity-based registration.

Recent studies began to use two-photon calcium imaging in real-time closed-loop experiments (Clancy et al., 2014; Hira et al., 2014; Prsa et al., 2017; Mitani et al., 2018). They require fast, real-time image processing, and lateral motion correction has not been implemented and ignored in most of these studies due to technical difficulties. However, imaging in awake animals necessarily includes motion artifacts, leading to many studies with *post hoc* analysis utilizing TurboReg and other image registration algorithms. Mitani et al. (2018) was the first to our knowledge to report real-time processing of calcium imaging incorporating lateral motion correction. This method used hill-climbing method to reduce computation of correlation coefficient between template and shifted images. Here, we report the details and the performance analysis of the implementation of fast motion correction, including some improvements we have made since the original study. In addition to real-time image processing, the method can also be used for faster *post hoc* processing.

After motion correction, typically ROIs are identified, and the relative change of the average fluorescence intensity of all the pixels in each ROI is calculated, based on the estimation of the baseline of the average fluorescence intensity. To estimate the baseline, percentile method and robust mean method are widely used, but each has shortcomings.

Percentile method estimates the baseline by taking a certain percentile of the fluorescence intensity time series. In calcium imaging, the calcium signal tends to have symmetric noise and sparse positive activity. Therefore, the percentile that represents the true baseline depends on the activity level. With no activity, the baseline should be the median of the distribution, while it corresponds to a lower percentile with more activity. In addition, when the specified percentile does not correspond to the true baseline, the amount of error depends on the noise level. With larger noise, the estimate is further away from the true baseline.

Another popular method for baseline estimation is robust mean. This method calculates mean of the signal while excluding outliers, which are assumed to be mainly from calcium activity. Outliers are typically defined as values different from the mean more than a set threshold, e.g., 2 standard deviation. An assumption for this method is that the mean is close to the true baseline, which is not the case when the activity level is high and can lead to poor baseline estimation.

To overcome these issues of the common methods, we estimated the baseline using kernel density estimation. Kernel density estimation is a method to estimate kernel density from a limited number of samples under the assumption that the kernel density function is smooth. Kernel density is a probability distribution from which each sample is produced. After the estimation, the peak of the kernel density approximates the center of the baseline. It only assumes that the baseline distribution peaks at the center with symmetric noise, and the peak is higher than the kernel density at fluorescence values during calcium events. Taking the peak of kernel density of a continuous distribution is comparable to taking the mode of a discrete distribution. Therefore, we hypothesized that there is little bias from increased activity and increased noise. Additionally, this method does not assume a specific distribution of noise and has fewer parameters than the other two methods. Here, we examined the three baseline estimation algorithms.

## Materials and Methods

### Experimental Methods

#### Animals

All procedures were in accordance with protocols approved by UCSD Institutional Animal Care and Use Committee and guidelines of the US National Institutes of Health. All animals were group housed in disposable plastic cages with standard bedding in a room on a reversed light cycle (12 h/12 h). Experiments were typically performed during the dark period. Cross between CaMK2a-tTA [JAX 003010] and tetO-GCaMP6s [JAX 024742] was used for cell body imaging. All the animals were C57bl/6 background.

#### Surgery

Surgical procedures were performed as previously described (Mitani et al., 2018). Adult mice (6 weeks or older, male and female) were anesthetized with isoflurane and injected with Baytril (10 mg/kg), dexamethasone (2 mg/kg) and buprenorphine (0.1 mg/kg) subcutaneously to prevent infection, inflammation and discomfort. A custom head-plate was glued and cemented to the skull. Craniotomy (∼3 mm) was performed over the right caudal forelimb area (300 μm anterior and 1,500 μm lateral from the bregma). A mixture of AAV1.Syn.Flex.GCaMP6f (1:5000–10000 final dilution) and AAV1.CMV.PI.Cre (1:2 final dilution) diluted in saline was injected 20–30 nL at 3–5 sites (∼250 μm depth, ∼500 μm apart) for dendrite imaging. Experiments were performed at least 7 days after surgery.

#### Imaging

Imaging was conducted with a commercial two-photon microscope (Bscope, Thorlabs) using a 16x objective (NIKON) with excitation at 925 nm (Ti-Sa laser, Newport). Images were acquired with ScanImage 4 (Vidrio Technologies). Imaging was conducted in awake animals. Images (512 × 512 pixels) were acquired at ∼28 Hz.

### Computational Methods

#### Motion Correction (General)

First, the template image was generated from 1000 image frames obtained before the experiments. Using the OpenCV template matching method (explained later), the first 500 images were aligned to the average of the last 500 images, the last 500 images were aligned to the average of the 500 corrected images, and the average of all the 1000 corrected images were used as a template.

Maximum absolute shift (m) in each direction was set to be 1/4 of the width (w) and height (h) of the image. From each edge of the template image, m pixels were cropped to take the central part [(w-2m) × (h-2m) pixels]. To correct motion artifact of each image, the objective is to find where in the image best matches this central part of the template and maximizes the correlation coefficient, which is used as a similarity metric (reviewed in Zitová and Flusser, 2003).

#### Motion Correction (Hill-Climbing Method)^3^

Instead of the global maximum, a local maximum can be reached iteratively by a hill-climbing technique (discussed in Lucas and Kanade, 1981). Let (x, y) be the current position, where the shift maximizes the correlation coefficient among all the shifts tested up to that point. The correlation coefficients for shifts (x+1, y), (x-1, y), (x, y+1), and (x, y-1) are calculated, and if there is any shift that increases the correlation coefficient, the current position is updated by 1 pixel to maximize the correlation coefficient. This step is repeated until the current position reaches the local maximum. To assess the computational complexity, we use big O notation here to indicate how the running time requirements grow as the input size grows. When an algorithm takes O(n) time for an input of size n, it means that the computation time scales linearly or less with n. Assuming the path is somewhat straight, the above hill climbing method requires O(m) steps to converge, and each step takes O(wh) time. Therefore, the computational complexity of the algorithm is O(mwh) under the assumption.

The complexity can be further reduced with a pyramid approach (Adelson et al., 1984). Aligning images downscaled by 2 takes 1/8 of the time, and the corresponding shift in the original image should give a good estimate. It may not give the exact maximum, but the difference should be small as long as the downscaled images have enough features for motion correction. Using this shift as an initial shift constrains the number of expected steps until the convergence to the final target. With a deep enough image pyramid, the computational complexity approaches O(wh). However, it requires spatial features for alignment to be available in all the downscaled images, and practically too deep a pyramid makes the algorithm unstable (Dubbs et al., 2016).

#### Motion Correction (Dense Search Method)^4^

To reach the global maximum, correlation coefficients for all possible shifts must be calculated. Naively implemented, computational cost of calculating correlation coefficient is proportional to the number of pixels, which is O(wh), and there are O(m^2^) potential shifts. Thus, the computational complexity of the naive algorithm is O(m^2^wh). This may be too slow to be applied to an image at the original resolution, but the time reduces rapidly as the image is further downscaled as it is proportional to the square of the number of pixels (note that m is proportional to w and h). We applied this method to estimate the optimal shift for the most downscaled image of the image pyramid, combined with the hill-climbing method at each scale as described above.

#### Motion Correction (OpenCV Template Matching Method)^5^

The objective is the same as the hill-climbing method, but with matchTemplate^6^ function of OpenCV^7^, it calculates correlation coefficient for every possible shift to reach the global maximum. The function uses discrete Fourier transform internally. Correlation theorem states that correlation coefficients can be efficiently calculated from Fourier transforms of images using Fast Fourier Transform (Brown, 1992). To increase the speed of computation, the image can be downscaled first, and then the shift can be multiplied for the original resolution, even though this would reduce the resolution of motion correction unless subpixel registration is applied.

#### Motion Correction (Subpixel Registration)

In many calcium imaging experiments, ROIs are small (∼10 pixels wide), and subpixel registration can improve the accuracy of calcium activity estimation. Subpixel registration has been done with optimization (Thévenaz et al., 1998) or upsampling (Guizar-Sicairos et al., 2008), but each has either speed or accuracy problem (Dubbs et al., 2016). Here, we used a parabola fit approach (Debella-Gilo and Kääb, 2011), which is faster and more suitable for real-time application. Subpixel registration was achieved by finding the peak of the correlation coefficient heatmap in subpixel accuracy using a parabola fit. In the hill-climbing method, it uses custom implementation in C++ using the 5 points in the heatmap (the peak and the adjacent points in 4 directions). In either *x*- or *y*-axis, correlation coefficient of the peak point and the two adjacent points are fit with a parabolic curve, and the peak of the parabolic curve was used as a subpixel estimate of the peak location in that axis. In the OpenCV method, the same algorithm implementation by William Arden (minMaxLocSubPix^8^) was used.

#### Performance Measurement of Motion Correction Methods

The objective of our methods is to maximize correlation coefficient between two images with lateral shifts. Motion correction algorithms by maximizing some similarity metric for lateral shifts have been widely used, e.g., TurboReg (Thévenaz et al., 1998) and moco (Dubbs et al., 2016), moco being especially close to our methods (moco minimizes L2 distance, and maximizing correlation coefficient is equivalent to minimizing L2 distance after normalization).

**FIGURE 1:**
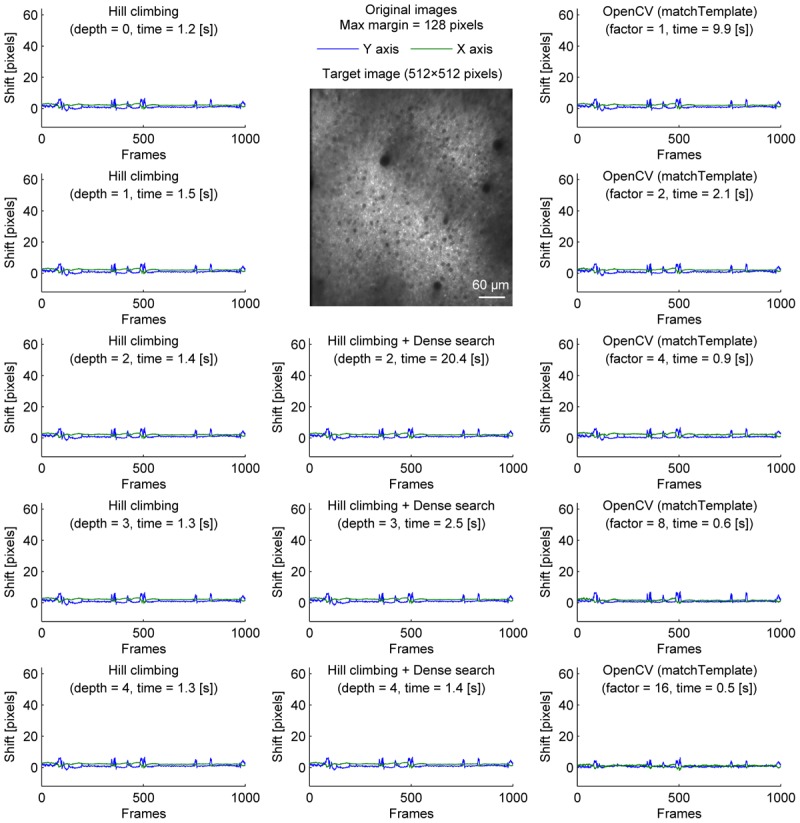
Comparison of speed and stability with different algorithms and parameters for neural ensemble imaging. Estimated shifts (blue: *Y* axis, green: *X* axis) with different algorithms and parameters for cell body imaging (512 × 512 pixels, 1000 frames). Dense search was slowest even at depth = 2 and shallower depth was not included.). Maximum shift for correction was set to be 128 pixels in all conditions.

To assess the accuracy of the algorithms, we examined how the correction is affected by adding random shifts (up to 16 pixels for neural ensemble imaging and up to 8 pixels for dendrite imaging in both *x*- and *y*-axis). For each frame, we repeatedly applied random shifts before motion correction for 100 times, and we examined the net translation, which is the sum of the initial random shift and the estimated translation. If an algorithm can correct initial random shifts along with motion artifacts, net translations should be consistent among different random shifts. If they are not consistent, it indicates that the algorithm has failed to align images. Here, frames with motion correction errors are defined as follows: among 100 random shifts, there are at least five net translations which are different from the median of the 100 net translations by more than 10 pixels. This was applied to the images in Figures 1, 3 with the algorithms and parameters examined in the figures. Chi-square test was used to compare the frequency of the frames with motion correction errors between different methods.

In addition, the speed of the algorithm applied to a 512 × 512 × 1000 movie was measured using the following configurations:

Hardware: Intel Core i7 4790, 16GB DDR3.

Software: Windows 10, Matlab R2014a, OpenCV 3.2.0, Visual Studio 2013.

#### Baseline Estimation

Three baseline estimation methods, percentile method, robust mean method and kernel density estimation method were examined on two consecutive windows of 2000 frames with and without apparent calcium activity. We used the difference of the estimates between two windows as a proxy for the sensitivity of the method to activity levels. First, the frames were split into 100 bins of 20 consecutive frames. For downscaling, the 20 frames were averaged. For downsampling, the first frame in each bin was chosen, and 19 frames were excluded. Downscaling reduces frame-by-frame noise by averaging, and downsampling simulates when the signal has more frame-by-frame noise. From these 100 values, percentile method uses the 20th percentile as a baseline estimate. For robust mean method, robust mean was calculated by excluding frames with absolute standard deviation larger than 2. This step was repeated until convergence using newly estimated robust mean and standard deviation at each step. Kernel density estimation method uses the peak of kernel estimation calculated by ksdensity function of Matlab (R2014a). Briefly, it is done by convolving a Gaussian kernel of width optimized to samples. Range of the estimates in four conditions (first and second window, downscaling and downsampling) was compared across the three methods.

In real-time experiments, every 20 frames, the baseline of each selected ROI was updated as follows. The preceding 2000 frames were used to estimate the baseline. 2000 frames were first split into 100 bins of 20 consecutive frames, and the average fluorescence of each bin was calculated. The baseline was estimated to be the value at the peak of the estimated kernel density distribution of the 100 average values. The script to process calcium intensity at each frame and estimate baseline is available at https://github.com/amitani/baseline_kde.

## Results

### Motion Correction

Especially in a real-time analysis application, time spent for motion correction should be kept short. While direct comparison to previously reported computation time is difficult, partly because the details of the configuration are often not reported, NoRMCorre was reported to correct a 512 × 512 × 2000 movie in 40 s (rigid transformation) and 117 s (non-rigid transformation) (Pnevmatikakis and Giovannucci, 2017). moco was reported to correct a 512 × 512 × 2000 movie in 90 sec, while TurboReg (Thévenaz et al., 1998) took 170 s (fast) and 298 s (slow) as reported in the same study (Dubbs et al., 2016). At 30 frames per second of image acquisition, 2000 frames take 66 s to capture. Only rigid transformation with NoRMCorre is slightly faster than this, but it still takes more than half of the acquisition time to process a frame. Considering the overhead of other steps, e.g., image transfer between processes and baseline estimation, it is necessary to make the processing time shorter for a real-time analysis.

We compared three implementations of motion correction based on correlation coefficient maximization. Our methods are similar to moco, but instead of using the whole overlap, we took the central part of the template image, and tried to maximize the correlation coefficient with the corresponding part of the target image. We first tested a hill-climbing method to find a local maximum. To increase speed, a pyramid approach was used. In this approach, the initial shift for hill-climbing is determined by the alignment of a downscaled image. Theoretically, it becomes faster with a deeper image pyramid, but there was no significant speed increase when the shift was small (Figure 1, left column). This is because the default initial shift (no shift) is close enough to the final shift.

**FIGURE 2:**
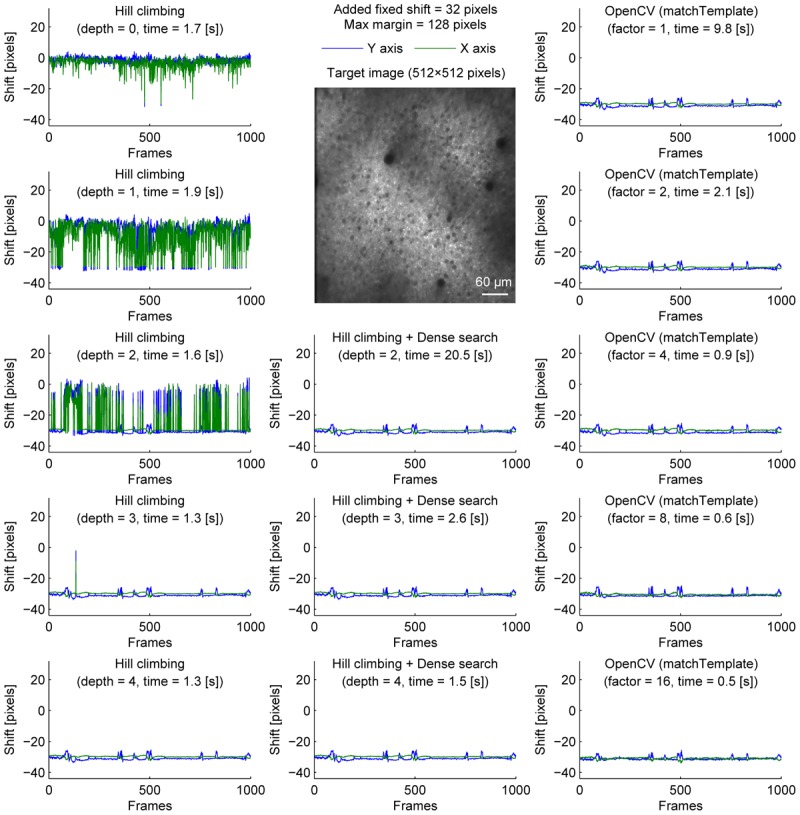
Comparison of speed and stability with different algorithms and parameters with artificially added fixed shifts for neural ensemble imaging. Same as Figure 1 with fixed shifts (32 pixels in both *X* and *Y* axis) added artificially before motion correction.

A caveat of the hill-climbing method is that it does not perform well when the true final shift is far from the initial shift. As the path becomes longer, it requires more computation, making it slower. Furthermore, if there is another local maximum along the path, the algorithm can converge to the local maximum, not the true final shift. This can be problematic in long experiments when a slow drift was not adjusted properly during the experiment. To simulate this situation where the images are far from the template, we artificially shifted each image frame by 32 pixels in both X and Y axis before motion correction (Figure 2). Without an image pyramid, the algorithm almost never converged to the true final shift; instead, it jumped among local maxima as indicated by sudden jumps in the corrected distance (Figure 2, top left). An image pyramid with four layers was required for the algorithm to converge to the same final shift estimated without the additional shift (Figure 2, left column). However, a deep image pyramid can lead to unstable results (see Discussions), and this solution may not be applicable in other situations.

To overcome this, we performed dense search to align the most downscaled image of an image pyramid, calculating correlation coefficients for all possible shifts to find the global maximum (Figure 2, middle column). With an image pyramid downscaling the image twice, this algorithm could converge to the same final shift as originally estimated without additional shift, different from the hill-climbing algorithm. However, it was implemented by naive exhaustive search, hindering the speed with shallow or no image pyramid.

Alternatively, we used matchTemplate function in OpenCV to search all possible shifts to reach the global maximum. When downscaling was used to reduce computation time, the estimated shift was transformed for the original image resolution with a parabola fit (no hill climbing was used). This was fast enough to apply to a 2× downscaled image, and more accurate than the hill-climbing method with similar speed.

**FIGURE 3:**
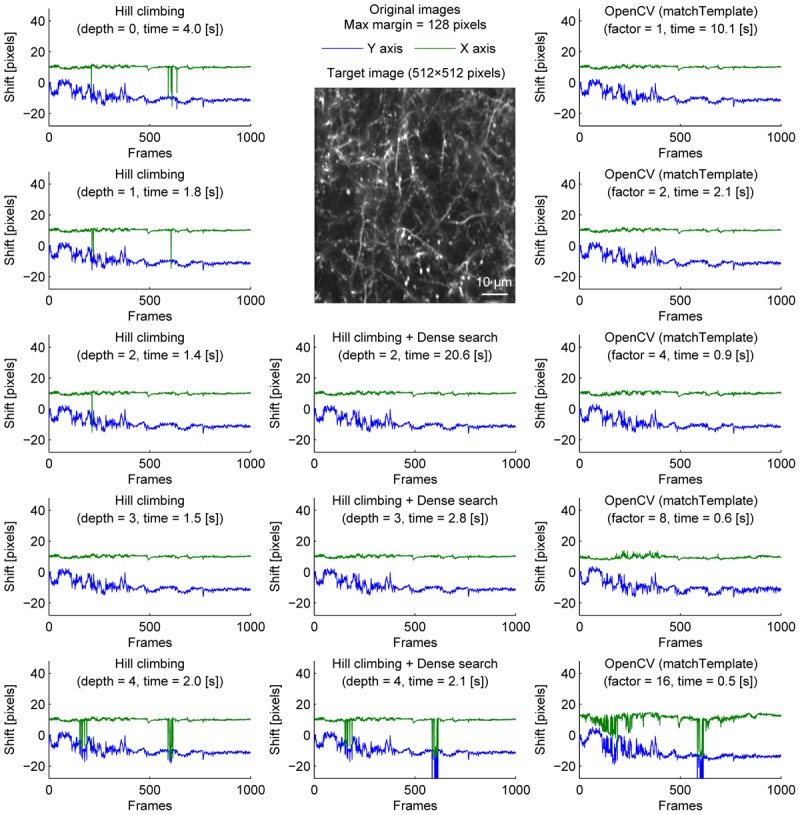
Comparison of speed and stability with different algorithms and parameters for dendrite imaging. Same as Figure 1 applied to dendrite imaging.

**FIGURE 4:**
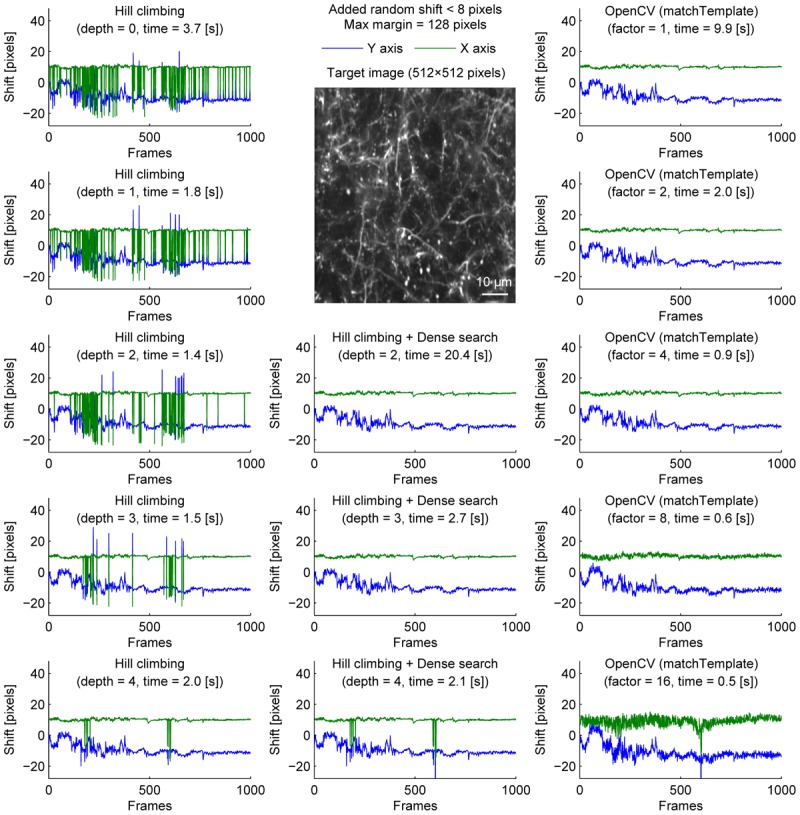
Comparison of speed and stability with different algorithms and parameters with artificially added random shifts for dendrite imaging. Same as Figure 1 applied to dendrite imaging with random shifts (up to 8 pixels in each direction) added artificially before motion correction. For visualization, these artificially added shifts were subtracted before the corrected distances are plotted.

We further examined the performance of the algorithms with sparsely labeled dendrite imaging data (Figure 3). This data is more challenging for motion correction because the motion artifact is severer with a higher zoom, and the signal tends to be weaker in dendrites. The results show that the alignment becomes unstable when the image is downscaled too much (Figure 3, bottom row), and the hill-climbing method is unstable without an image pyramid or with a shallow image pyramid (Figure 3, left column). To simulate severer motion artifacts, we added random shift up to 8 pixels in each direction at each frame (Figure 4). The results further illustrate the speed and the stability of OpenCV template matching method in severer conditions. Interestingly, the estimate becomes noisier with OpenCV template matching method when applied to more downscaled images. This is because the estimate is affected by how the pixels are split into patches for downscaling. Note that this effect is negligible when the image is only downscaled up to a factor of 2.

To provide one example set of quantifications of the accuracy of the shown algorithms, we examined the number of frames with motion correction errors (Methods). For neural ensemble imaging (from Figure 1), a hill-climbing method with an image pyramid of 4 layers, dense search methods with image pyramids of 2, 3, and 4 layers, and OpenCV methods with downscaling factors of 1, 2, 4, 8, and 16 had no frames with motion correction errors. On the contrary, hill-climbing methods with 0, 1, 2, and 3 layers of image pyramids had 1000 (*p* < 0.0001, Chi-square test, comparison to methods with no errors; same applies hereafter), 881 (*p* < 0.0001), 266 (*p* < 0.0001), and 4 (*p* = 0.0453) frames, respectively. For dendrite imaging (from Figure 3), dense search methods with 2 and 3 layers of image pyramids, and OpenCV methods with downscaling factors of 1, 2, 4, and 8 had no frames with motion correction errors. Hill-climbing methods with 0, 1, 2, 3, and 4 layers of image pyramids had 829 (*p* < 0.0001), 728 (*p* < 0.0001), 423 (*p* < 0.0001), 173 (*p* < 0.0001), and 74 (*p* < 0.0001) frames with motion correction errors, respectively, and a dense search method with an image pyramid of 4 layers and an OpenCV method with a downscaling factor of 16 had 78 (*p* < 0.0001) and 32 (*p* < 0.0001) frames with motion correction errors, respectively. These results support that with large shifts the hill-climb methods fail to find the global maximum by converging to a local maximum, and that motion correction becomes unstable if the images are too downscaled.

**FIGURE 5:**
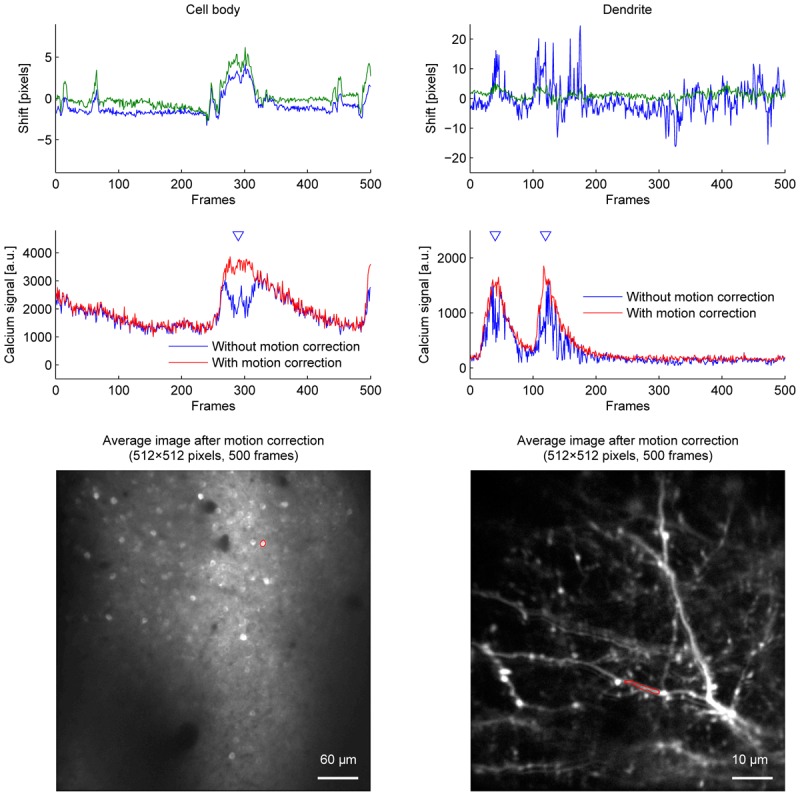
The effect of motion correction on calcium fluorescence signal. Estimated shifts (blue: *Y* axis, green: *X* axis) **(top row)**, calcium fluorescence signal **(middle row)**, and average images **(bottom row)** of cell body imaging **(left column)** and dendrite imaging **(right column)**. Calcium fluorescence signal was estimated without motion correction (blue lines) and with motion correction (red lines). Blue triangles indicate when motion correction improves signals. ROIs for calcium fluorescence signals are outlined in red on the average images. OpenCV method with twice downscaled images was used for motion correction.

Among the algorithms and parameters examined in the current study, we conclude that applying the OpenCV method to a twice downscaled image best balances speed and accuracy in our experiments. This was the fastest combination which did not cause motion correction errors even when the downscaling factor was either doubled or quadrupled. Figure 5 shows the improvement of fluorescence signals from motion correction.

### Baseline Estimation

**FIGURE 6:**
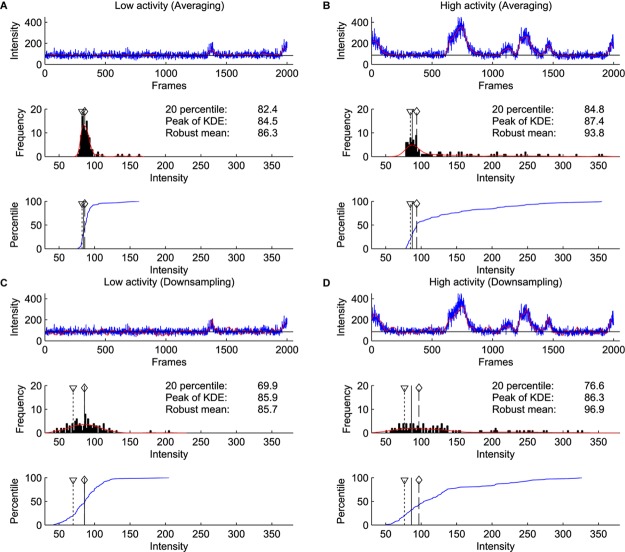
Comparison of percentile, robust mean, and kernel density estimate methods for baseline estimation. **(A)** Analysis on a window of 2000 frames with little calcium activity. Blue line shows raw signal, and red line shows the signal after binning and averaging every 20 frames, which was used for baseline estimation (Top). Histogram of signal intensity. Red line indicates kernel density estimate. A triangle with dotted line indicates the 20th percentile, a solid line indicates the peak of kernel density estimate, a diamond with a dashed line indicates robust mean (excluding values not within 2 SD from the mean) (Middle). Cumulative density function of the intensity distribution (blue line) (Bottom). **(B)** Same as **(A)** on the next 2000 frames to **(A)**. This window contains a high level of calcium activity. **(C,D)** Same as **(A,B)** with downsampling. Red line indicates the signal downsampled by 20 (excluding 19 frames from 20 frames), which is further used for baseline estimation (Top). Note that only the kernel density estimate method identified consistent baseline values in averaged and downsampled data.

We compared the baseline estimation on two consecutive windows of 2000 frames with and without apparent calcium activity. We used the difference of the estimates between two windows as a proxy for the sensitivity of the method to activity levels. Furthermore, to simulate increased noise, we compared downsampling and averaging of the bins (Figure 6). These results show that the estimates by the kernel density estimation method were the most robust across different conditions, even with increased activity and increased noise.

### Implementation of Real-Time Image Processing for Closed-Loop Experiments

We developed a real-time image processing pipeline for two-photon calcium imaging for closed-loop experiments that includes a lateral motion correction with a comparable accuracy to popular *post hoc* methods as well as an improved baseline estimation method. In the pipeline, each image is first copied to a memory-mapped file at the time of acquisition by a custom plugin^9^ for ScanImage 4. A custom Qt GUI application^10^ reads each image from the memory-mapped file, corrects for motion artifact with the OpenCV template matching method, and saves the corrected image in another memory-mapped file. Another instance of Matlab reads the corrected image from the memory-mapped file, calculates average pixel intensity of each ROI, estimates baseline^11^, and calculates ΔF/F. This information of relative change in fluorescence is further used in a closed-loop experiment.

## Discussion

Here, we discussed our implementation of fast motion correction and baseline estimation algorithms for real-time image processing of two-photon calcium imaging. This is to our knowledge the first reported real-time image processing pipeline not specific to a particular imaging platform for closed-loop experiments with motion artifact correction. The source code of the implementation is hosted as public repositories at GitHub. We included a plugin for ScanImage, and it would also work with other Matlab-based imaging platforms with minimal modifications. An earlier version of the implementation was used in a previous study for real-time feedback experiments (Mitani et al., 2018).

Our implementation of motion correction is significantly faster than previously reported software packages, while maintaining the accuracy by globally maximizing correlation coefficient. Using a regular personal computer, our OpenCV template matching method with downscaling by a factor of two processed a 512 × 512 × 1000 movie in less than 3 s, while moco (Dubbs et al., 2016) and NoRMCorre (Pnevmatikakis and Giovannucci, 2017) (rigid) are reported to take 40 and 90 s to process a 512 × 512 × 2000 movie, respectively.

Images are often downscaled to reduce computational cost for motion correction, e.g., image pyramid method in TurboReg (Thévenaz et al., 1998), but the process can erase fine spatial features necessary for motion correction. Supporting this, a previous study reported that an image pyramid method downscaling images 3 and 4 times gave severe errors (Dubbs et al., 2016). We observed such errors in dendrite imaging with a deep image pyramid. On the other hand, due to iterative optimization process, the hill-climbing method with a shallow image pyramid converged to a local maximum and failed when the shift is large. These imply that the number of layers of an image pyramid has to be properly adjusted for each experimental condition. However, parameter tuning often incorporates trials and errors, which is not suitable for real-time experiments. In contrast, our OpenCV-based implementation is fast enough to find a global maximum in real-time without extensive downscaling.

Out methods and moco estimate translation of the whole image. However, non-uniform artifact can arise from deformation of tissue and distortion due to a finite scanning speed (Pnevmatikakis and Giovannucci, 2017). Deformation is more problematic with a larger imaging field, and distortion is more problematic when movements while scanning a frame correspond to more pixels. In such applications, non-rigid correction can be beneficial. NoRMCorre is a non-rigid registration method based on piecewise-rigid algorithm, which involves translation-based registration of patches (Pnevmatikakis and Giovannucci, 2017). While NoRMCorre is not fast enough to process 512 × 512 images at 28 Hz, combining our method with their piecewise-rigid algorithm may lead to faster non-rigid motion correction applicable to real-time processing.

Baseline estimation of calcium signal is another crucial step of calcium imaging. Among percentile method, robust mean method and kernel density method, we showed that our kernel density method gave most consistent estimate among different noise and activity levels. Furthermore, a potential limitation specific to the percentile method may arise when there is increasing or decreasing trend of the baseline. Typically, a percentile lower than the median is used as estimated baseline. In this case, there is a jitter in timing caused by the changing trend of the baseline. When the baseline is increasing, the increase of the estimate happens later. On the other hand, when the baseline is decreasing, the decrease of the estimate happens earlier. For example, let us consider a situation estimating baseline from 100 values without noise and activity. In this case, the 20th percentile of the intensity values is at the 20th bin when the baseline is constantly increasing, and at the 80th bin when the baseline is constantly decreasing. This creates temporal difference equivalent to 60 bins. According to the increasing or decreasing trend of the baseline, there is a different degree of delay in the estimate of the baseline. Robust mean method and kernel density method do not have this shortcoming.

Kernel density method has only two parameters, the length of the window and the size of each bin, which can be adjusted depending on the signal to noise ratio, the activity duration, and how quickly and how often the baseline fluctuates. Binning and averaging are commonly performed preprocessing steps in other methods as well, while other methods have extra parameters, e.g., percentile value for percentile method, and cutoff threshold for robust mean method. Having less parameters is especially useful in closed-loop experiments, when rerunning the analysis with updated parameters is not possible.

Our implementation of fast motion correction and baseline estimation provides a real-time image processing pipeline, and the small number of parameters to tune makes it easy to use and assess for each application. The pipeline can also be used for fast *post hoc* analysis. Indeed many of the recent publications from our laboratory used this or previous versions of the method for *post hoc* analysis (Chu et al., 2016, 2017; Hwang et al., 2017; Peters et al., 2017; Li et al., 2018; Mitani et al., 2018), showing the applicability of this method across different brain areas, different expression methods and different imaging configurations.

## Author Contributions

AM and TK conceived the project and wrote the paper. AM performed the experiments and software development. All authors contributed to manuscript revision, read and approved the submitted version.

## Conflict of Interest Statement

The authors declare that the research was conducted in the absence of any commercial or financial relationships that could be construed as a potential conflict of interest.

## Footnotes

1 https://scanbox.org/2014/12/09/using-auto-stabilization/

2 https://github.com/saghayee/Tracking-based-registration

3 https://github.com/amitani/mex_tools/tree/master/mexBilinearRegistrator

4 https://github.com/amitani/mex_tools/tree/master/mexBilinearRegistrator

5 https://github.com/amitani/mex_tools/tree/master/cvMotionCorrect

6 https://docs.opencv.org/3.2.0/de/da9/tutorial_template_matching.html

7 https://opencv.org/

8 http://answers.opencv.org/question/29665/getting-subpixel-with-matchtemplate/

9 https://github.com/amitani/mex_tools

10 https://github.com/amitani/onlineMotionCorrection

11 https://github.com/amitani/baseline_kde

## References

[B1] Adelson E. H. Anderson C. H. Bergen J. R. Burt P. Ogden J. (1984). Pyramid methods in image processing. *RCA Eng.* 29 33–41.

[B2] Aghayee S. Winkowski D. E. Bowen Z. Marshall E. E. Harrington M. J. Kanold P. O. (2017). Particle tracking facilitates real time capable motion correction in 2D or 3D two-photon imaging of neuronal activity. *Front. Neural Circuits* 11:56. 10.3389/fncir.2017.00056 28860973 PMC5559509

[B3] Brown L. G. (1992). A survey of image registration techniques. *ACM Comput. Surv.* 24 325–376. 10.1145/146370.146374

[B4] Chu M. W. Li W. L. Komiyama T. (2016). Balancing the robustness and efficiency of odor representations during learning. *Neuron* 92 174–186. 10.1016/j.neuron.2016.09.004 27667005 PMC5061050

[B5] Chu M. W. Li W. L. Komiyama T. (2017). Lack of pattern separation in sensory inputs to the olfactory bulb during perceptual learning. *eNeuro* 4 ENEURO.287–17. 10.1523/ENEURO.0287-17.2017 28955724 PMC5615249

[B6] Clancy K. B. Koralek A. C. Costa R. M. Feldman D. E. Carmena J. M. (2014). Volitional modulation of optically recorded calcium signals during neuroprosthetic learning. *Nat. Neurosci.* 17 807–809. 10.1038/nn.3712 24728268 PMC4361947

[B7] Debella-Gilo M. Kääb A. (2011). Sub-pixel precision image matching for measuring surface displacements on mass movements using normalized cross-correlation. *Remote Sens. Environ.* 115 130–142. 10.1016/j.rse.2010.08.012

[B8] Dombeck D. A. Khabbaz A. N. Collman F. Adelman T. L. Tank D. W. (2007). Imaging large-scale neural activity with cellular resolution in awake, mobile mice. *Neuron* 56 43–57. 10.1016/j.neuron.2007.08.003 17920014 PMC2268027

[B9] Dubbs A. Guevara J. Yuste R. (2016). moco: fast motion correction for calcium imaging. *Front. Neuroinform.* 10:6. 10.3389/fninf.2016.00006 26909035 PMC4754735

[B10] Greenberg D. S. Kerr J. N. (2009). Automated correction of fast motion artifacts for two-photon imaging of awake animals. *J. Neurosci. Methods* 176 1–15. 10.1016/j.jneumeth.2008.08.020 18789968

[B11] Guizar-Sicairos M. Thurman S. T. Fienup J. R. (2008). Efficient subpixel image registration algorithms. *Opt. Lett.* 33 156–158. 10.1364/OL.33.000156 18197224

[B12] Hira R. Ohkubo F. Masamizu Y. Ohkura M. Nakai J. Okada T. (2014). Reward-timing-dependent bidirectional modulation of cortical microcircuits during optical single-neuron operant conditioning. *Nat. Commun.* 5:5551. 10.1038/ncomms6551 25418042

[B13] Hwang E. J. Dahlen J. E. Mukundan M. Komiyama T. (2017). History-based action selection bias in posterior parietal cortex. *Nat. Commun.* 8:1242. 10.1038/s41467-017-01356-z 29089500 PMC5663966

[B14] Li W. L. Chu M. W. Wu A. Suzuki Y. Imayoshi I. Komiyama T. (2018). Adult-born neurons facilitate olfactory bulb pattern separation during task engagement. *eLife* 7:e33006. 10.7554/eLife.33006 29533179 PMC5912906

[B15] Lucas B. D. Kanade T. (1981). “An Iterative Image Registration Technique with an Application to Stereo Vision (IJCAI),” in *Proceedings of the 1981 DARPA Image Understanding Workshop*, (Washington, DC).

[B16] Mitani A. Dong M. Komiyama T. (2018). Brain-Computer interface with inhibitory neurons reveals subtype-specific strategies. *Curr. Biol.* 28 77–83.e4. 10.1016/j.cub.2017.11.035 29249656 PMC5760288

[B17] Peters A. J. Lee J. Hedrick N. G. O’Neil K. Komiyama T. (2017). Reorganization of corticospinal output during motor learning. *Nat. Neurosci.* 20 1133–1141. 10.1038/nn.4596 28671694 PMC5656286

[B18] Pnevmatikakis E. A. Giovannucci A. (2017). NoRMCorre: an online algorithm for piecewise rigid motion correction of calcium imaging data. *J. Neurosci. Methods* 291 83–94. 10.1016/j.jneumeth.2017.07.031 28782629

[B19] Prsa M. Galiñanes G. L. Huber D. (2017). Rapid integration of artificial sensory feedback during operant conditioning of motor cortex neurons. *Neuron* 93 929–939.e6. 10.1016/j.neuron.2017.01.023 28231470 PMC5330804

[B20] Thévenaz P. Ruttimann U. E. Unser M. (1998). A pyramid approach to subpixel registration based on intensity. *IEEE Trans. Image Process.* 7 27–41. 10.1109/83.650848 18267377

[B21] Zitová B. Flusser J. (2003). Image registration methods: a survey. *Image Vis. Comput.* 21 977–1000. 10.1016/S0262-8856(03)00137-9

